# Influence of Nb and Mo Substitution on the Structure and Magnetic Properties of a Rapidly Quenched Fe_79.4_Co_5_Cu_0.6_B_15_ Alloy

**DOI:** 10.3390/ma16186288

**Published:** 2023-09-19

**Authors:** Lukasz Hawelek, Przemyslaw Zackiewicz, Anna Wojcik, Jacek Hudecki, Tymon Warski

**Affiliations:** 1 Lukasiewicz Research Network—Institute of Non-Ferrous Metals, 5 Sowinskiego St., 44-100 Gliwice, Poland; przemyslaw.zackiewicz@imn.lukasiewicz.gov.pl (P.Z.); tymon.warski@imn.lukasiewicz.gov.pl (T.W.); 2 Institute of Metallurgy and Materials Science, Polish Academy of Sciences, 25 Reymonta St., 30-059 Krakow, Poland; wojcik.a@imim.pl; 3 Laryngology Department, School of Medicine in Katowice, Medical University of Silesia in Katowice, 20-24 Francuska St., 40-027 Katowice, Poland; jacek.hudecki@gmail.com; 4 Faculty of Mechanical Engineering, PhD School, Silesian University of Technology, 18A Konarskiego St., 44-100 Gliwice, Poland

**Keywords:** soft magnetic materials, materials characterisation, toroidal cores, crystal structure

## Abstract

The importance of amorphous and nanocrystalline Fe-based soft magnetic materials is increasing annually. Thus, characterisation of the chemical compositions, alloying additives, and crystal structures is significant for obtaining the appropriate functional properties. The purpose of this work is to present comparative studies on the influence of Nb (1, 2, 3 at.%) and Mo (1, 2, 3 at.%) in Fe substitution on the thermal stability, crystal structure, and magnetic properties of a rapidly quenched Fe_79.4_Co_5_Cu_0.6_B_15_ alloy. Additional heat treatments in a vacuum (260–640 °C) were performed for all samples based on the crystallisation kinetics. Substantial improvement in thermal stability was achieved with increasing Nb substitution, while this effect was less noticeable for Mo-containing alloys. The heat treatment optimisation process showed that the least lossy states (with a minimum value of coercivity below 10 A/m and high saturation induction up to 1.7 T) were the intermediate state of the relaxed amorphous state and the nanocomposite state of nanocrystals immersed in the amorphous matrix obtained by annealing in the temperature range of 340–360 °C for 20 min. Only for the alloy with the highest thermal stability (Nb = 3%), the α-Fe(Co) nanograin grows, without the co-participation of the hard magnetic Fe_3_B, in a relatively wide range of annealing temperatures up to 460 °C, where the second local minimum in coercivity and core power losses exists. For the remaining annealed alloys, due to lower thermal stability than the Nb = 3% alloy, the Fe_3_B phase starts to crystallise at lower annealing temperatures, making an essential contribution to magneto-crystalline anisotropy, thus the substantial increase in coercivity and induction saturation. The air-annealing process tested on the studied alloys for optimal annealing conditions has potential use for this type of material. Additionally, optimally annealed Mo-containing alloys are less lossy materials than Nb-containing alloys in a frequency range up to 400 kHz and magnetic induction up to 0.8 T.

## 1. Introduction

Although amorphous and nanocrystalline soft magnetic materials have been studied for many years, they are constantly being developed and underestimated. The (Fe, Co)-based alloys have constituted an important group of soft magnetic materials where applications for high magnetic flux densities are required (data storage, pole tips for high-field magnets, and transformers) [1,2]. Their magnetic properties are tailored by compositional variation and structure induced by the annealing process [3,4]. The combination of superior soft magnetic properties, low power losses, and high saturation magnetisation has fueled interest in Fe-based nanocrystalline materials. Today, Finemet alloys have excellent soft magnetic properties [5], while Nanoperm alloys were developed to achieve high saturation magnetisation [6]. Moreover, by introducing Co to Nanoperm, Hitperm alloys were developed to increase the Curie temperature [7]. Many other studies have shown that a small amount of Co-doping coupled with appropriate annealing is an effective method of improving the saturation magnetisation of many Fe-based alloys [8,9,10,11]. Co leads to the alignment of Fe moments, thus increasing the atomic magnetic moment of individual Fe atoms [12]. Unfortunately, high Co addition deteriorates soft magnetic properties due to its large magneto-crystalline anisotropy, which is in accordance with the Slater-Pauling curves [13,14]. 

Cu is insoluble in Fe-based alloys and can refine the primary particles and promote the uniformity of grain dispersion. In Fe-based alloys, during the annealing process, Cu atoms agglomerate in clusters and act as nucleation sites for the α-Fe particles. The crystallisation behaviour of Cu-free alloys is completely different from that of Cu-containing alloys. Additionally, the appropriate amount of Cu addition can optimise and allow the highest possible magnetic properties [15,16]. The co-presence of Nb and Cu can further refine the microstructure. Nb promotes the Cu cluster’s nucleation on a much finer scale, impacts the grain size, and hinders the growth of the α-Fe particles [17].

Additionally, 3% Nb is the maximum limit of effective grain refining [18]. For Si-containing alloys, Mo and W are less effective than Nb in limiting grain growth [19]. Both elements (Mo and Nb) are good grain growth inhibitors in Si-containing alloys [20,21]. Moreover, both elements provide improved GFA, thermal stability, and a shift to higher temperatures at the beginning of the alpha-Fe phase crystallisation [22,23,24]. For this reason, they are crucial to obtaining amorphous materials and enabling proper, controlled processing. However, Mo and Nb are paramagnetic elements and magnetically affect the local atomic environment around Fe. A larger addition of these elements may negatively affect the magnetic properties of the alloy (e.g., saturation induction); hence, it is not recommended to use < 5 at.% Mo or Nb for materials that require a high Bs value [25,26,27]. It is worth noting that, currently, a large proportion of commercially available Fe-based metallic ribbons contain the addition of Nb to obtain the appropriate structure and magnetic properties. 

Zhu et al. reported the possibility of partial Nb replacement by Mo in Fe_80_(Nb_1-x_Mo_x_)B_15_ alloys, which improved the material’s thermal stability and magnetic properties. Similar work was carried out by Ramasamy et al., comparing the effect of Mo and Nb addition on Fe_37.5_Co_37.5_B_20_Si_5_ alloy in the form of rods, showing that at 4 at.% Mo addition, the material has better soft magnetic properties and retains similar thermal stability as the material with Nb addition. However, a further increase in Mo reduced the magnetic and thermal properties [25]. Therefore, it is necessary to study the effect of minor additions of Nb and Mo on the magnetic properties, thermal stability, and crystallisation kinetics of the Si-free Fe-B metallic ribbons.

This work comparatively studies the influence of Nb and Mo additions on the B-rich Fe_79.4_Co_5_Cu_0.6_B_15_ alloy. The effect of the increase in the amount of grain refining elements (Nb and Mo) for Fe up to 3 at.% on magnetic properties, such as the B(H) relationship, core losses, and complex magnetic permeability as a function of annealing temperature during the conventional isothermal annealing process, is evaluated. For optimised heat-treated conditions, the crystal structure is then verified by X-ray diffraction and transmission electron microscopy. This information provides deeper insight into the impact of grain refining on comprehensive magnetic properties.

## 2. Materials and Methods

The amorphous alloys with nominal composition Fe_79.4-x_Co_5_M_x_Cu_0.6_B_15_, M = Nb, Mo, x = 1, 2, 3 at.% in the form of a ribbon with a thickness of approximately 18–23 µm and a width of 5–7 mm were obtained via the melt spinning technique on a 650 mm diameter Cu wheel in an air atmosphere. The primary alloys were produced from pure chemical elements (Fe (3N), Co (3N), and Cu (3N)) and FeNb_65_ (2.5N) and FeB_18_ (2.5N) alloys using the induction furnace SecoWarwick VIM-LAB 50–60 (SecoWarwick S.A., Swiebodzin, Poland). For the annealing process and subsequent magnetic measurements, the amorphous ribbon was wound into toroidal cores with an inner diameter of ~20 mm and an outer diameter of ~30 mm. The toroidal cores were isothermally annealed for 20 min in a vacuum furnace (10^−3^ mbar) at different temperatures (260–640 °C) to achieve the nanocrystalline state. The structural properties of the as-spun and heat-treated ribbons were studied by X-ray diffraction (XRD). XRD measurements were performed at room temperature using a Rigaku MiniFlex 600 diffractometer (Rigaku Co., Tokyo, Japan) equipped with CuK_α_ radiation (λ = 0.1542 nm), K_β_ Ni filter, and the D/teX Ultra-high-speed silicon strip detector. The crystallisation processes were monitored using differential scanning calorimetry (DSC) performed with a 10 °C/min heating rate using the thermal analyser Netzsch DSC 214 Polyma (NETZSCH, Houston, TX, USA). Transmission electron microscopy (TEM) images in the bright-field (BF) mode and selected area diffraction patterns (SADPs) were recorded for the selected annealed samples using the Tecnai G2 F20 (200 kV) electron microscope (FEI, Hillsboro, OR, USA). The Remacomp C-1200 (MAGNET-PHYSIK Dr. Steingroever GmbH) magnetic measurement system was used to determine hysteresis and magnetic properties (saturation induction Bs, coercivity Hc, core power losses P_10/50,_ i.e., in B = 1 T and f = 50 Hz) of the annealed samples. Additionally, for samples annealed at optimum conditions, the Ps parameter was measured in the frequency range f = 50 Hz–400 kHz and the magnetic induction B = 0.1–0.8 T. For samples annealed at characteristic temperatures, the complex magnetic permeability at room temperature in the frequency range f = 10^4^–10^8^ Hz was determined using the Agilent 4294A impedance analyser (Agilent, Santa Clara, CA, USA).

## 3. Results and Discussion

**Figure 1:**
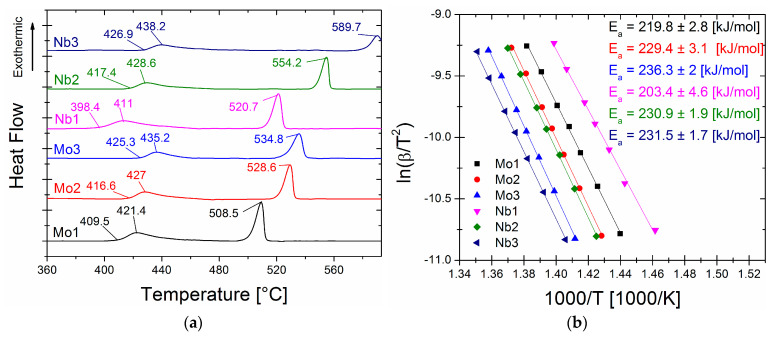
(**a**) DSC signals for as-spun alloys. (**b**) Kissinger plots with calculated activation energy for as-spun alloys.

The X-ray diffraction method verified the amorphousness of the quenched as-spun ribbons; all recorded patterns possessed only diffused maxima characteristics for a fully amorphous state. Figure 1a depicts the DSC curves of the as-spun alloys, showing that the crystallisation of all ribbons proceeded in two stages: primary crystallisation of α-Fe(Co) and secondary Fe-B phase crystallisation. The onset of the primary crystallisation peak (T_x1_) value and both crystallisation peaks (T_p1_, T_p2_) were marked in the figure, while all crystallisation temperatures with the temperature interval (ΔT_x_ = T_x2_ − T_x1_) were gathered in Table 1. Nb and Mo for Fe substitution increased the primary crystallisation temperature (T_x1_) with substitution content. However, the temperature interval (ΔT_x_) increased substantially for Nb-containing alloys. The effect of Mo substitution on ΔT_x_ was lower than for Nb. This indicates that Nb substitution might enhance the thermal stability of the alloy matrix, which is beneficial for crystallisation heat treatment. 

The kinetics of α-Fe type phase crystallisation (primary crystallisation peak) were studied using the DSC method by performing measurements with heating rates ranging from 10 to 50 °C/min. The Kissinger model [28] was used to determine the average activation energies for such a non-isothermal crystallisation process. This method is based on Equation (1):

(1) $$ln\left(\frac{\phi }{T_{p}^{2}}\right)=ln\left(\frac{A_{0}R}{E_{a}}\right)-\frac{E_{a}}{\left(RT_{p}\right)},$$

where ϕ is the heating rate, $T_{p}$ is the temperature of the crystallisation peak, $E_{a}$ is the activation energy, *R* is the gas constant, and $A_{0}$ is the pre-exponential factor. By linear fitting of $ln\left(\phi /T_{p}^{2}\right)$ vs. $1/T_{p}$ curves, the average activation energy *E_a_* of the process was determined from the slopes of these curves. The Kissinger plots and calculated *E_a_* values are presented in Figure 1b. The *E_a_* values correlated positively with T_x1_ for the Mo-containing ribbons, where *E_a_* increased with T_x1_ from 219.8 kJ/mol for Mo = 1% up to 236.3 kJ/mol for Mo = 3%. There was some peculiar behaviour for the Nb-containing alloys, where ribbons with Nb = 2% and Nb = 3% *E_a_* values were similar (*E_a_* = 231 kJ/mol), while there were no changes in the T_x1_ increase. Calculated *E_a_* values, as an extension of the values obtained for Fe_85_B_15_ (199.1 kJ/mol) and Fe_84.6_Cu_0.6_B_15_ (223.4 kJ/mol) alloys, fit into the obtained trend of values [29].

**Figure 2:**
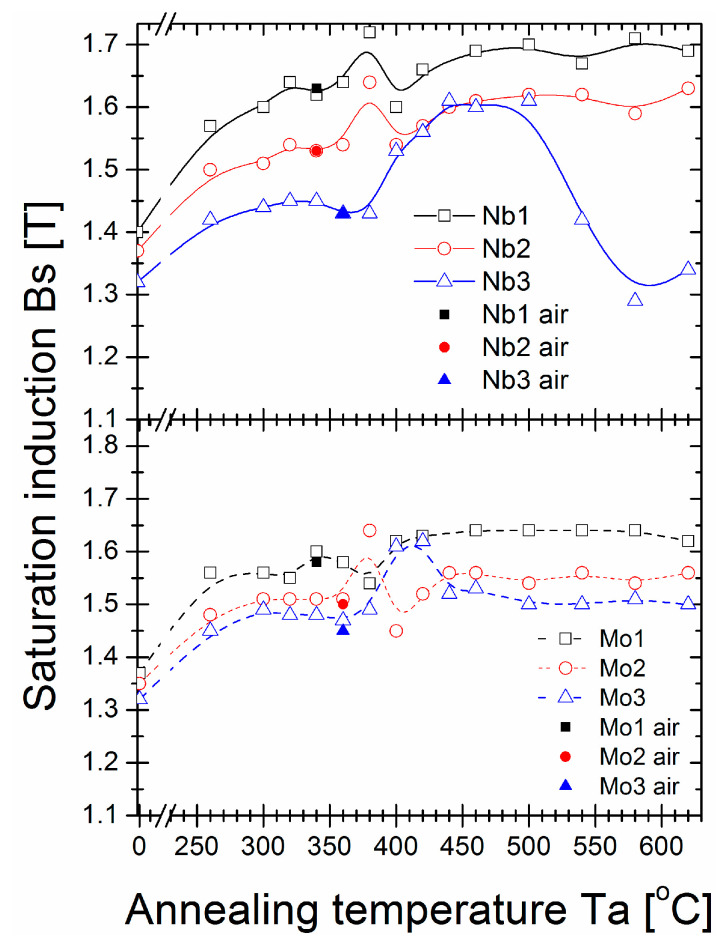
Saturation induction from annealing temperature dependence.

**Figure 3:**
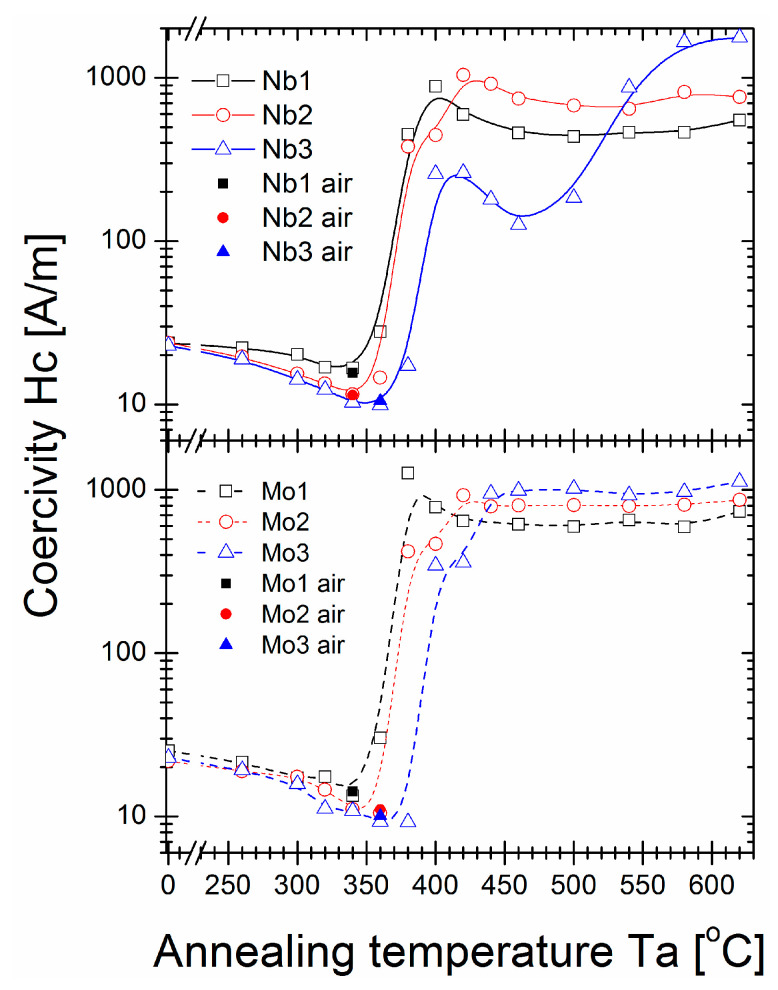
Coercivity from annealing temperature dependence.

**Figure 4:**
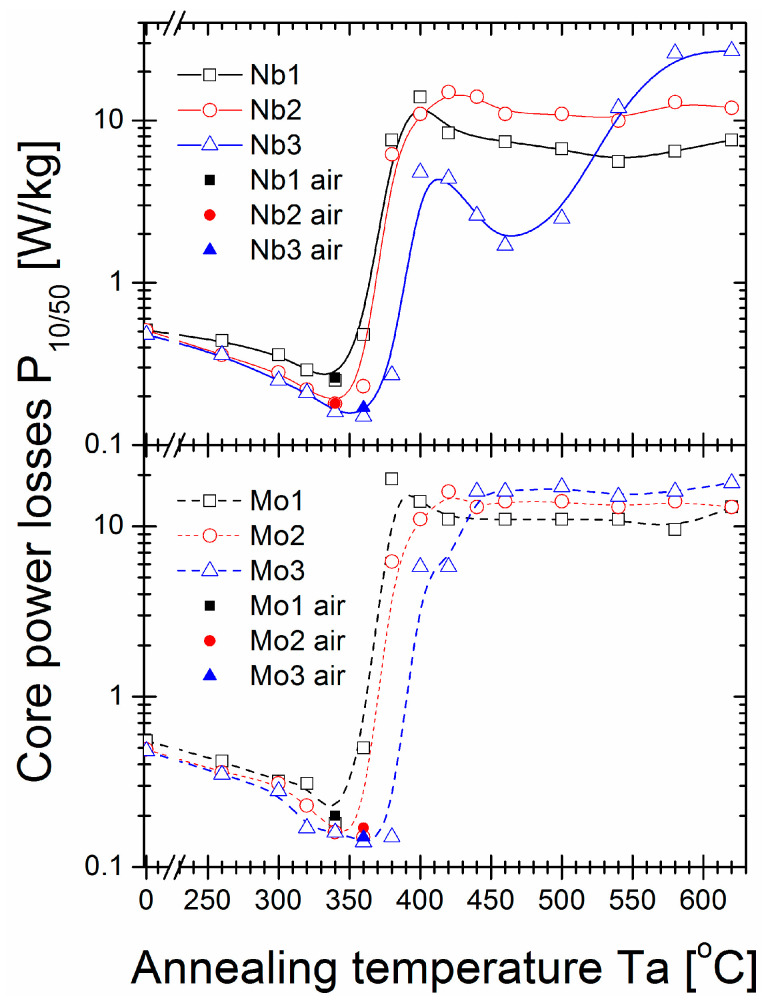
Core power losses from annealing temperature dependence.

The saturation induction Bs (Figure 2), coercivity Hc (Figure 3), and core power losses P_10/50_ (Figure 4) of Nb- and Mo-containing alloys were assessed after annealing for 20 min at varying annealing temperatures (Ta). For as-quenched alloys, saturation induction decreased from 1.4 T to 1.3 T with Nb content, which can be attributed to the weaker ferromagnetic exchange coupling. Mo-containing alloys did not exhibit such an effect, and Bs was equal to 1.35 T for all studied samples. The Bs values of all alloys (except an Nb = 3% alloy) increased with increasing Ta, with noticeable fluctuations in the 350–420 °C temperature range. The maximum value of induction saturation for all samples was detected for annealed alloys with a Ta over 400 °C. The maximum induction saturation value reached ~1.7 T for Nb = 1% alloy and ~1.6 T for Nb = 2% and Nb = 3% alloys. However, the Nb = 3% alloy had a significant drop in Bs values for Ta > 500 °C, usually as a deterioration effect. For Mo-containing alloys, a rather flat plateau occurred in Bs(Ta > 420 °C) dependence, and Bs decreased with Mo content from ~1.64 T for Mo = 1% to ~1.56 T for Mo = 2% and ~1.53 T for Mo = 3%. This Bs(Ta) behaviour is completely different from previously studied Fe_85_B_15_ and Fe_84.6_Cu_0.6_B_15_ alloys, where the saturation induction deteriorated substantially just over the temperature value of optimal annealing (at 330 °C) [29]. Herein, for Nb and Mo-containing alloys, such a high induction region was broadened to 600 °C. Regarding Hc(Ta) and P_10/50_(Ta) (Figure 3 and Figure 4, respectively) dependences, a large increase in both values (log scale) was observed in the same Ta region (350–420 °C) where Bs values fluctuated. Additionally, for the Nb = 3% alloy, a strong “double-minima shape” dependence was identified, while for other alloys, this dependence shape was rather weak (in log-scale) or unidentified, like for Mo = 1% and Mo = 2% alloys. The global minimum of the Hc and P_10/50_ values were identified for Ta = 340 °C (for Nb = 1%, Nb = 2%, and Mo = 1% alloys) and Ta = 360 °C (for the remaining alloys). The least lossy alloy content was obtained for 3% Nb or Mo with P_10/50_ = 0.15 W/kg and P_10/50_ = 0.14 W/kg, respectively. For the Nb = 3% alloy second-local minimum in Hc(Ta) and P_10/50_(Ta), dependencies existed for Ta = 460 °C. However, Hc and P_10/50_ were approximately ten times greater. By comparing with the previously studied Fe_85_B_15_ and Fe_84.6_Cu_0.6_B_15_ alloys, the Nb- and Mo- addition extended the relatively narrow thermal stability window of the optimal annealing process; however, the Hc(Ta) and Ps(Ta) first minima had similar values and shapes as the minima for Fe_85_B_15_ and Fe_84.6_Cu_0.6_B_15_ alloys [29]. For optimal annealing conditions defined by Ta with a global minimum P_10/50_ value, an additional test for the air-annealing process was performed. As shown in Figure 2, Figure 3 and Figure 4, results presented for magnetic properties confirmed that all study materials were sufficiently resistant to oxygen content during annealing in the temperature range of 340–360 °C for 20 min. This seems to be a cheaper, less demanding alternative manufacturing process option that may be used on an industrial scale. 

**Figure 5:**
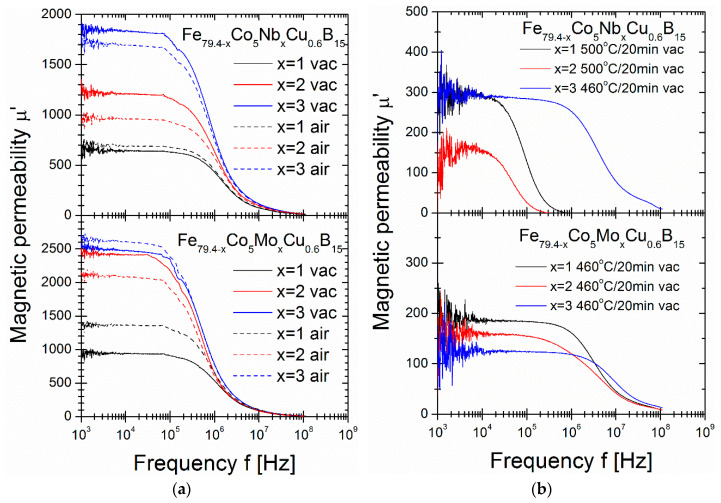
(**a**) Magnetic permeability for air- and vacuum-annealed samples under P_10/50_ optimum conditions. (**b**) Magnetic permeability for vacuum-annealed samples at a second-local minimum of P_10/50_(Ta) dependence.

Figure 5 shows the µ’ of the alloy complex permeability at two different stages: in optimal annealing conditions at Ta = 340 °C and Ta = 360 °C in a vacuum and air (Figure 5a) and for annealing close to the second-local minimum of P_10/50_(Ta) dependence at 460 and 500 °C in a vacuum (Figure 5b). Figure 5a shows that vacuum-annealed Mo and Nb samples influence µ’, increasing from 631 for Nb = 1% alloy to 1746 for Nb = 3% alloy and from 907 for Mo = 1% alloy to 2543 for Mo = 3% alloy. For air-annealed samples, similar increases in µ’ were observed for Nb, while the Mo content effect was similar with small differences in values. The magnetic permeability of vacuum-annealed alloys at higher temperatures (Figure 5b) shows a significant drop in values up to µ’ = 100–300 for all samples. Similar effects have previously been observed for Fe_85_B_15_ and Fe_84.6_Cu_0.6_B_15_ alloys [29]. Additionally, the abrupt change in the µ’ slopes shifts into lower frequency values for Nb-containing alloys. These frequencies are identified from maximum µ’ values, designated as the cut-off frequency (f_cut-off_), and strongly depend on the crystal structure (grain size and phase content). The µ’ values, together with the rest of the magnetic parameter values, are presented in Table 2. For the interpretation of all the magnetic property fluctuations, it is necessary to verify the crystal structure at both states: optimal annealing temperature (340–360 °C) and second-local minimum (460–500 °C). 

**Figure 6:**
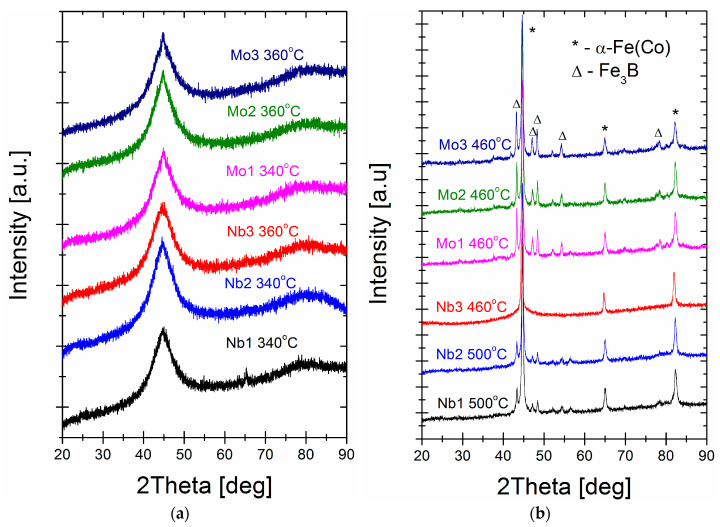
XRD patterns for annealed alloys: (**a**) at P_10/50_ optimum conditions; (**b**) at a second-local minimum of P_10/50_(Ta) dependence.

**Figure 7:**
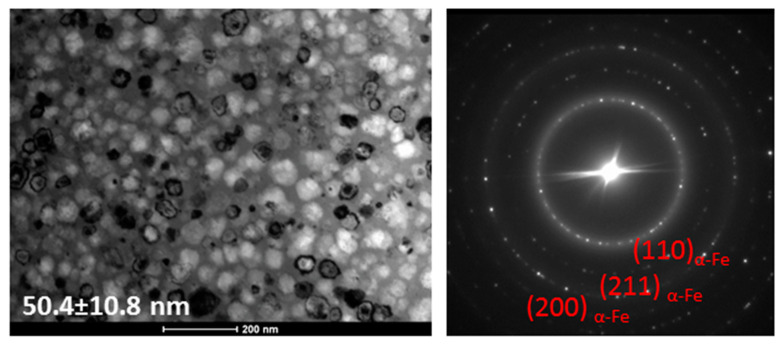
Set of BF image and SAED pattern for vacuum-annealed Fe_76.4_Co_5_Nb_3_Cu_0.6_B_15_ at 460 °C for 20 min.

From inspection of the XRD patterns for the optimal annealing temperature (Figure 6a), the initial state of the α-Fe(Co) phase crystallisation with the dominant contribution of the amorphous state as an amorphous diffraction halo. Only a small diffraction peak emerging from the first-order amorphous halo at 2 Theta = 43–45 deg is seen. The crystal structure of annealed alloys at higher temperatures (Figure 6b) shows an almost fully crystalline two-phase (α-Fe(Co) + Fe_3_B) system structure for all alloys except the Nb = 3% alloy, where only the α-Fe(Co) phase exists as a dominant contribution with a small amount of amorphous diffused content. A bright-field (BF) image and selected area electron diffraction (SAED) pattern are shown in Figure 7. It can be seen from the BF TEM image that nanoscale grains precipitate randomly, and the residual amorphous matrix is the minority component. Statistical analysis and careful grain size determination using the Gatan Digital Microscopy suite helped to determine the average nanograin size of ~50 nm. The SAED pattern indicated that only the α-Fe(Co) phase existed with randomly oriented nanocrystals. TEM observations agree with the XRD results presented in Figure 6. Based on structural studies and thermal analysis, we can interpret the origin of the changes in magnetic properties. The α-Fe(Co) phase is identified here as the soft magnetic phase, while the Fe_3_B phase belongs to the hard magnetic counterpart [30,31]. During the annealing process in the temperature range of 340–380 °C, the atoms rearrange locally in a short-range order and form clusters immersed in the amorphous matrix. The coupling of these clusters leads to magnetocrystalline anisotropy. With increased annealing temperature, the mean grain size decreases, and only for the Nb = 3% alloy, the crystallisation products remain the same up to 460–500 °C. The ferromagnetic exchange between the α-Fe(Co) nanocrystals is enhanced, as seen in the increasing value of saturation induction in Figure 2. A further increase in the annealing temperature causes further grain coarsening, hard magnetic Fe_3_B phase precipitation, and a strong drop in the Bs(Ta) dependence. This effect of stably coarsening the α-Fe(Co) phase is only possible thanks to the high thermal stability ΔT_x_ of 155 °C shown in the DSC studies. For all other alloys with lower thermal stability, the hard magnetic Fe_3_B phase co-precipitates with α-Fe(Co), blocking the stable α-Fe(Co) coarsening. According to the random anisotropy model proposed by Herzer [32], the Hc is proportional to the fourth power of magneto-crystalline anisotropy. As previously reported, this value for the Fe_3_B phase is equal to 430 kJ/m3 and is substantially larger than for the α-Fe(Co) phase [33]. High-induction soft magnetic materials are especially used in high-frequency applications where the core power losses of the materials are significant. Thus, for samples annealed at optimum conditions, the Ps parameters were measured in the frequency range f = 50 Hz–400 kHz and the magnetic induction B = 0.1–0.8 T. Table 3 presents results for four different f and B measurement combinations: 50 Hz/0.8 T, 50 kHz/0.8 T, 100 kHz/0.4 T, and 400 kHz/0.1 T. The core power losses decrease with increasing Mo and Nb content. Moreover, the Mo-containing alloys are ~20% less lossy than the Nb-containing ones, which agrees with the change tendency of the coercivity values. 

## 4. Conclusions

In this work, comparative studies on the influence of Nb (1, 2, 3 at.%) and Mo (1, 2, 3 at.%) for Fe substitution on the thermal stability, crystal structure, and magnetic properties of a rapidly quenched Fe_79.4_Co_5_Cu_0.6_B_15_ alloy were performed. The research results can be summarised as follows:

- Successive Nb substitution enhances thermal stability more efficiently than Mo, which is beneficial for crystallisation heat treatment.
- The 20-min vacuum-annealing process was optimised for a wide temperature range from 260 to 640 °C. For the Nb = 3% alloy, the highest thermal stability favours the precipitation of only α-Fe(Co) nanograins in the amorphous matrix at a broadened annealing temperature range of up to 460 °C. Hc(Ta) and P_10/50_(Ta) dependences correlate strongly with crystal structure evolution. For example, the α-Fe(Co) grain growth and Fe_3_B phase precipitation significantly increase the magneto-crystalline anisotropy, leading to an increase in the Hc value. A double minimum of Hc(Ta) dependence exists with different α-Fe(Co) nanograin contents. The first minimum is related to a relaxed amorphous state, while the second is related to a relaxed α-Fe(Co)/amorphous nanocomposite.
- For all alloys except Nb = 3%, the limited thermal stability of <150 °C does not only allow for α-Fe(Co) nanograins to precipitate at higher temperatures. Rather, the fast co-precipitation of the hard magnetic Fe_3_B phase also substantially increases magnetic saturation while stabilising Hc and P_10/50._
- Optimally vacuum-annealed alloys (when the P_10/50_ is at a minimum) exhibit excellent magnetic properties with high saturation induction up to 1.7 T and low coercivity below 10 A/m. Optimally vacuum-annealed Mo-containing alloys are 20% less lossy than Nb-containing alloys in the whole B (up to 0.8 T) and f (up to 400 kHz) ranges.
- The air-annealing process may be an alternative and effective heat treatment process for use on an industrial scale. There are no significant differences in magnetic properties between the vacuum- and air-annealed materials under optimal annealing conditions.

## Figures and Tables

**Table 1:** Characteristic crystallisation temperatures: T_p1_, T_p2,_ T_x1_, T_x2,_ ΔT_x_.

Composition	T_x1_ [°C]	T_p1_ [°C]	T_x2_ [°C]	T_p2_ [°C]	ΔT_x_ = T_x2_ − T_x1_ [°C]
Fe_78.4_Co_5_Nb_1_Cu_0.6_B_15_	398.4	411	513	520.7	114.6
Fe_77.4_Co_5_Nb_2_Cu_0.6_B_15_	417.4	428.6	546.4	554.2	129
Fe_76.4_Co_5_Nb_3_Cu_0.6_B_15_	426.9	438.2	581.6	589.7	154.7
Fe_78.4_Co_5_Mo_1_Cu_0.6_B_15_	409.5	421.4	500.3	508.5	90.8
Fe_77.4_Co_5_Mo_2_Cu_0.6_B_15_	416.6	427	521	528.6	104.4
Fe_76.4_Co_5_Mo_3_Cu_0.6_B_15_	425.3	435.2	525.5	534.8	100.2

**Table 2:** Magnetic properties for selected heat-treated samples (vac—vacuum-annealing, air—air-annealing).

Composition	Ta [°C]	Bs [T]	H_c_ [A/m]	P_10/50_[W/kg]	µ’	f_cut-off_ [kHz]
Fe_78.4_Co_5_Nb_1_Cu_0.6_B_15_	340 vac	1.62	16.7	0.25	631	1472
	340 air	1.63	15.6	0.26	678	1394
	500 vac	1.7	437	6.7	287	78
Fe_77.4_Co_5_Nb_2_Cu_0.6_B_15_	340 vac	1.53	11.5	0.18	1165	1022
	340 air	1.53	11.4	0.18	932	1472
	500 vac	1.62	675	11	150	45
Fe_76.4_Co_5_Nb_3_Cu_0.6_B_15_	360 vac	1.43	9.9	0.15	1746	791
	360 air	1.43	10.5	0.17	1614	791
	460 vac	1.6	126	1.7	291	3951
Fe_78.4_Co_5_Mo_1_Cu_0.6_B_15_	340 vac	1.6	13.4	0.18	907	1182
	340 air	1.58	14.2	0.2	1306	867
	460 vac	1.64	615	11	187	3351
Fe_77.4_Co_5_Mo_2_Cu_0.6_B_15_	360 vac	1.51	10.5	0.15	2416	519
	360 air	1.5	11	0.17	1904	483
	460 vac	1.56	801	14	159	3950
Fe_76.4_Co_5_Mo_3_Cu_0.6_B_15_	360 vac	1.47	9.3	0.14	2543	529
	360 air	1.45	10.1	0.15	2681	483
	460 vac	1.53	989	16	119	8993

**Table 3:** Core losses Ps measured at selected f and B.

Composition	Ta [°C]	50 Hz/0.8 T [W/kg]	50 kHz/0.8 T [W/kg]	100 kHz/0.4 T [W/kg]	400 kHz/0.1 T [W/kg]
Fe_78.4_Co_5_Nb_1_Cu_0.6_B_15_	340 vac	0.0025	15	13	7.3
Fe_77.4_Co_5_Nb_2_Cu_0.6_B_15_	340 vac	0.0018	13	11	6.6
Fe_76.4_Co_5_Nb_3_Cu_0.6_B_15_	360 vac	0.0015	11	8.8	4.7
Fe_78.4_Co_5_Mo_1_Cu_0.6_B_15_	340 vac	0.0018	12	10	5.5
Fe_77.4_Co_5_Mo_2_Cu_0.6_B_15_	360 vac	0.0014	9.1	7.6	5.0
Fe_76.4_Co_5_Mo_3_Cu_0.6_B_15_	360 vac	0.0012	7.4	5.9	3.6

## Data Availability

The data presented in this study are available on request from the authors.
